# Die digitalen Fortschrittshubs Gesundheit – Gemeinsame Datennutzung über die Universitätsmedizin hinaus

**DOI:** 10.1007/s00103-024-03883-9

**Published:** 2024-05-16

**Authors:** Dagmar Krefting, Udo Bavendiek, Joachim Fischer, Gernot Marx, Denise Molinnus, Torsten Panholzer, Hans-Ulrich Prokosch, Ines Leb, Jens Weidner, Martin Sedlmayr

**Affiliations:** 1 Institut für Medizinische Informatik, Universitätsmedizin Göttingen, Von-Siebold-Straße 3, 27075 Göttingen, Deutschland; 2 Klinik für Kardiologie und Angiologie, Medizinische Hochschule Hannover, Hannover, Deutschland; 3 Medizinische Fakultät Mannheim, Zentrum für Präventivmedizin und Digitale Gesundheit, Universität Heidelberg, Mannheim, Deutschland; 4 Klinik für Operative Intensivmedizin und Intermediate Care, Universitätsklinikum Aachen, Aachen, Deutschland; 5 Institut für Medizinische Biometrie, Epidemiologie und Informatik, Universitätsmedizin Mainz, Mainz, Deutschland; 6 Lehrstuhl für Medizinische Informatik, Friedrich-Alexander-Universität Erlangen-Nürnberg, Erlangen, Deutschland; 7 Institut für Medizinische Informatik und Biometrie, Technische Universität Dresden, Dresden, Deutschland

**Keywords:** Digitalisierung, Medizin, Datenintegration, Interoperabilität, Behandlungspfad, Intersektorale Gesundheitsversorgung, Digitalization, Medicine, Data integration, Interoperability, Patient journey, Intersectoral healthcare

## Abstract

Die digitalen Fortschrittshubs Gesundheit pilotieren die Erweiterbarkeit der Konzepte und Lösungen der Medizininformatik-Initiative für eine Verbesserung der regionalen Gesundheitsversorgung und -forschung. Die 6 geförderten Projekte adressieren dabei unterschiedliche Erkrankungen, Stationen in der regionalen Gesundheitsversorgung und Methoden der institutionsübergreifenden Datenverknüpfung und -nutzung. Trotz der Verschiedenheit der Szenarien und regionalen Voraussetzungen sind die technischen, regulativen und organisatorischen Herausforderungen und Hürden, auf die die Fortschrittshubs bei der konkreten Implementierung der Lösungen treffen, oft ähnlich. Daraus ergeben sich teilweise gemeinsame Lösungsansätze, teilweise aber auch Forderungen an die Politik, die über das aus Sicht der Fortschrittshubs begrüßenswerte Gesundheitsdatennutzungsgesetz hinausgehen.

In diesem Beitrag stellen wir die digitalen Fortschrittshubs vor und diskutieren Erreichtes, Herausforderungen und Lösungsansätze, die eine gemeinsame Nutzung von Daten aus den Universitätskliniken und den nichtakademischen Institutionen des Gesundheitssystems ermöglichen und auch nachhaltig zu einer Verbesserung von medizinischer Versorgung und Forschung beitragen können.

## Abstract (en)

The digital health progress hubs pilot the extensibility of the concepts and solutions of the Medical Informatics Initiative to improve regional healthcare and research. The six funded projects address different diseases, areas in regional healthcare, and methods of cross-institutional data linking and use. Despite the diversity of the scenarios and regional conditions, the technical, regulatory, and organizational challenges and barriers that the progress hubs encounter in the actual implementation of the solutions are often similar. This results in some common approaches to solutions, but also in political demands that go beyond the Health Data Utilization Act, which is considered a welcome improvement by the progress hubs.

In this article, we present the digital progress hubs and discuss achievements, challenges, and approaches to solutions that enable the shared use of data from university hospitals and non-academic institutions in the healthcare system and can make a sustainable contribution to improving medical care and research.

## Einleitung

Die Medizininformatik-Initiative (MII) hat in der *Aufbau- und Vernetzungsphase*, die von 2018 bis 2022 vom Bundesministerium für Bildung und Forschung (BMBF) gefördert wurde, zahlreiche Lösungen zur gemeinsamen Nutzung der klinischen Behandlungsdaten der Universitätskliniken entwickelt [1]. Herausforderungen waren und sind dabei zum einen die organisatorische, technische und regulative Harmonisierung der Prozesse des Datenteilens als auch die Implementierung der FAIR-Richtlinien für die Daten selber, also medizininformatische Methoden und Standards zur Auffindbarkeit, Erreichbarkeit, Interoperabilität und Nachnutzbarkeit der Daten [2]. Wesentliche Ergebnisse sind eine abgestimmte breite Patienteneinwilligung als Grundlage der Datenbereitstellung, national einheitliche Nutzungsbedingungen und Vertragswerke sowie ein national abgestimmter Datenstandard – der sogenannte MII-Kerndatensatz [3, 4]. Durch das Forschungsdatenportal Gesundheit können nun strukturierte stationäre Behandlungsdaten der Universitätskliniken standortübergreifend gefunden, beantragt und analysiert werden [5].

**Abb. 1:**
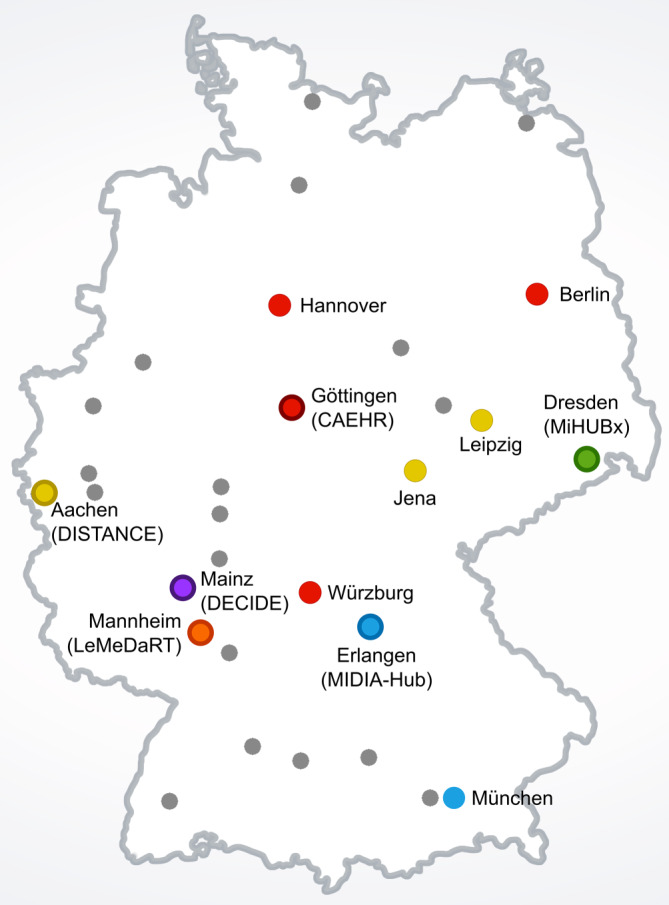
Universitätskliniken, die an den Digihubs beteiligt sind. Der koordinierende Standort ist jeweils mit dem Namen des Digihubs gekennzeichnet. Weitere beteiligte universitäre Standorte sind farblich zugeordnet. Die regionalen Partner können aufgrund ihrer Vielfalt nicht mit abgebildet werden, sie sind aber online in den jeweiligen Projektbeschreibungen zu finden. Quelle: eigene Abbildung

Es existieren jedoch Beschränkungen dieses Ansatzes: Viele Behandlungspfade starten weder im Universitätsklinikum noch enden sie dort. Der stationäre Aufenthalt ist oft eine Episode im Behandlungsverlauf und für das Verständnis, die Diagnose und eine optimierte Therapie einer Krankheit sind Gesundheitsdaten entlang des gesamten Patientenpfades sowie eine gute Abstimmung der Akteur:innen der regionalen Gesundheitsversorgung (Abb. 1) in der Regel hochrelevant. Hier setzt das Fördermodul der digitalen Fortschrittshubs an: In der Ausschreibung des BMBF vom 28.02.2020 – also kurz vor Ausbruch der Corona-Pandemie in Deutschland – heißt es [6]: „Eine besondere Herausforderung wird es daher sein, Patientendaten, die an nicht-universitären medizinischen Einrichtungen entstehen, für die Gesundheitsforschung nutzbar zu machen. … Ziel ist die Erprobung der Machbarkeit einer forschungskompatiblen, sektorenübergreifenden Datenbereitstellung in der medizinischen Praxis der regionalen Versorgung sowie die modellhafte Überprüfung des Mehrwerts für Patientinnen und Patienten, medizinisches Fachpersonal und die Wissenschaft.“

Die darauffolgenden Jahre der Pandemie haben allen Beteiligten, inklusive der breiten Öffentlichkeit, eindrücklich gezeigt, wie groß die Herausforderungen der Gesundheitsdatennutzung in Deutschland sind [7–10]. Die digitalen Fortschrittshubs Gesundheit (Digihubs) versuchen hier beispielhaft Lösungen zu entwickeln und durch bessere Datenverfügbarkeit und -nutzung konkrete Verbesserungen zu erreichen.

Im Folgenden stellen wir die 6 Digihubs mit ihren jeweiligen Anwendungsfällen, regionalen Akteur:innen und Lösungsansätzen kurz vor (Tab. 1, Abb. 1, 2 und 3). Anschließend diskutieren wir gemeinsame Erkenntnisse aus der ersten Halbzeit der Projekte sowie Vorschläge, wie in den aktuellen Entwicklungen der Nutzen der Digihubs in den konkreten Anwendungsfällen, aber auch darüber hinaus nachhaltig gesichert werden kann, um Erkenntnisse und Innovationen der medizinischen Datenwissenschaften in die Praxis zu bringen.

**Tab. 1:** Digitale Fortschrittshubs Gesundheit (Digihubs): Akteur:innen, Erkrankungsbilder, Regionen und Methoden

Name	Außeruniversitäre Akteur:innen	Erkrankungsbilder bzw. medizinische Bereiche	Regionen	Methoden
‍CAEHR	FA, PAT, RD, RH, RK	Schlaganfall, Transkatheter-Aortenklappenimplantation, Herzinsuffizienz, koronare Herzkrankheit	Berlin, Mainfranken, Südniedersachsen	Apps, ePA, PROMs, Portal, Telekonsil, Wearables
‍DECIDE	FA, PAT, RH	Lungen- und Dickdarmkrebs, Depressionen, sportmedizinische Prä- und Rehabilitation	Mainz, Rheinland-Pfalz	Apps, ePA, Telekonsil, Telemedizin, Wearables
‍DISTANCE	PAT, RK, FA, HA	Intensivmedizinische Behandlung	Nordrhein-Westfalen Thüringen-Sachsen	Apps, PROMs
‍LeMeDaRT	FA, HA, RH	Krebserkrankungen, Lebererkrankungen, Respiratorische Infektionen	Schwarzwald	Telemedizin
‍MIDIA-HUB	FA, PAT	Brust- und Prostatakrebs, multiple Sklerose	Erlangen-Nürnberg München	eKA, Portal
‍MiHUBx	RK	Diabetische Augenerkrankung, Krebserkrankungen, regionales Gesundheitsmonitoring	Sachsen	eKA, Telekonsil, Portal

*eKA* elektronische Krankenakte, *ePA* elektronische Patientenakte, *FA* Facharztpraxen, *HA* Hausarztpraxen, *PAT* Patient:innen, *PROMs* Patient-Reported Outcome Measures (Patientenfeedbackmethode), *RD* Rettungsdienst, *RH* Rehaeinrichtungen, *RK* regionale Krankenhäuser

**Abb. 2:**
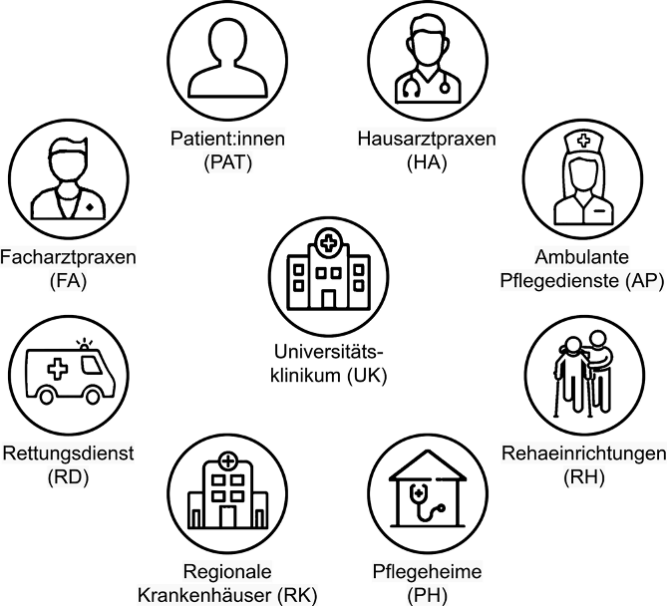
Akteur:innen in der regionalen Gesundheitsversorgung. *Quelle*: eigene Abbildung

**Abb. 3:**
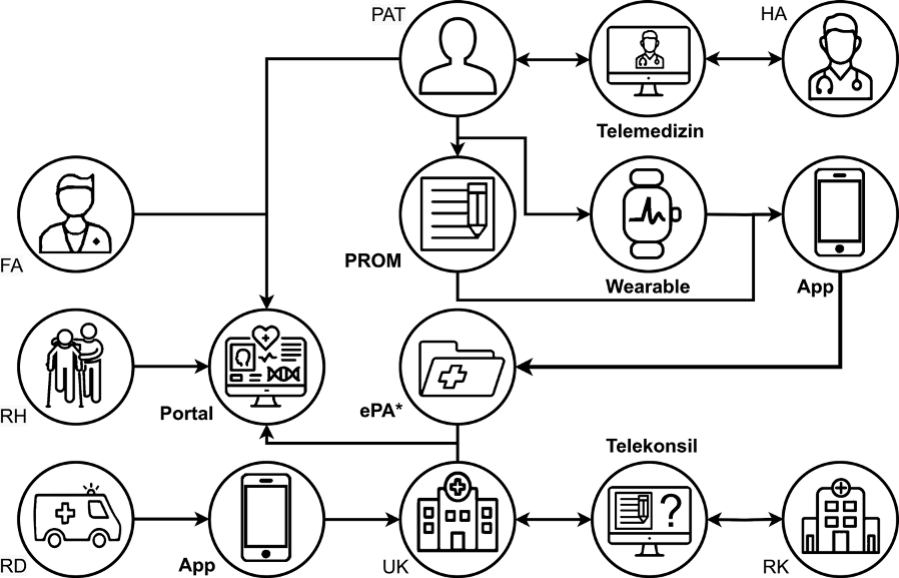
Digitale Methoden und Werkzeuge der digitalen Fortschrittshubs. *Quelle*: eigene Abbildung. ePA elektronische Patientenakte, *FA* Facharztpraxen, *HA* Hausarztpraxen, *PAT* Patient:innen, *PROM* Patient-Reported Outcome Measure (Patientenfeedbackmethode), *RD* Rettungsdienst, *RH* Rehaeinrichtungen, *RK* regionale Krankenhäuser, *UK* Universitätsklinikum

## Die digitalen Fortschrittshubs

### CAEHR: Verbesserung der Versorgung von Herz-Kreislauf-Patient:innen

Herz-Kreislauf-Erkrankungen stellen trotz wichtiger Fortschritte in der Behandlung noch immer die häufigste Todesursache in Deutschland dar [11]. Darüber hinaus nehmen diese Erkrankungen oftmals einen chronischen Verlauf. Sowohl die betroffenen Patient:innen als auch die behandelnden Ärzt:innen müssen sich auf eine dauerhafte sowie individuelle und personalisierte Behandlung einstellen. Das Grundkonzept von CAEHR („CArdiovascular diseases – Enhancing Healthcare through cross-sectoral Routine data integration“) ist die Verbesserung relevanter Schnittstellen vom Universitätsklinikum zu vorhergehenden oder nachfolgenden Stationen im Behandlungspfad. Je eine Schnittstelle, ein Erkrankungsbild und eine Region bilden dabei einen der 3 Anwendungsfälle:

1. Notfallversorgung bei Schlaganfall in der Region Mainfranken,
2. Rehabilitation nach Transkatheter-Aortenklappenimplantation in der Metropolregion Niedersachsen,
3. fachärztliche Versorgung bei Herzinsuffizienz und koronarer Herzkrankheit in Berlin.

Dabei sollte eine forschungskompatible elektronische Gesundheitsakte – basierend auf openEHR als persistentes Datenmodell und HL7-FHIR als Transferstandard – in allen Anwendungsfällen zum Einsatz kommen. Hier baut CAEHR auf umfangreichen Vorarbeiten aus dem Use Case Kardiologie des HiGHmed-Konsortiums auf [12, 13]. Ein zentraler Aspekt ist die Evaluation der Maßnahmen. Vor der technischen Implementierung wurden deshalb mit den Stakeholdern Evaluationskriterien entwickelt und abgestimmt, um den Mehrwert für Patient:innen, Gesundheitsdienstleistende und Forschende messen zu können [14, 15]. Die Ergebnisevaluation wird im Prä-Post-Design durchgeführt, dazu laufen in allen 3 Anwendungsfällen bereits die entsprechenden Studien.

Ein weiterer Fokus betrifft die Nachhaltigkeit. Aufgrund der aktuell hohen Dynamik in den rechtlichen Rahmenbedingungen von der Einführung einzelner Anwendungen der Telematikinfrastruktur (TI) 2.0 bis zu Gesundheitsdatennutzungsgesetz und europäischen Regelungen zu Medizinprodukten, künstlicher Intelligenz und Cyberresilienz, aber auch der schnellen technischen Entwicklungen liegt der Fokus auf technologieunabhängigen Konzepten und Spezifikationen. Der bereits in der Konzeption berücksichtigte Roll-out der Anwendungsfälle in jeweils eine andere Region soll zeigen, wie sich die Lösungen unter anderen rechtlichen, organisatorischen und technischen Rahmenbedingungen implementieren lassen.

### DECIDE: Erhöhung der Versorgungsqualität bei Krebs und psychischen Erkrankungen in strukturschwachen Gebieten

Die Versorgungsqualität bei Krebs und psychischen Erkrankungen ist in strukturschwachen Gebieten eine Herausforderung, da beide Krankheitsbilder nach der Diagnose oft eine komplexe Therapiefindung und langfristige Therapiebegleitung benötigen. Eine engmaschige fachärztliche Betreuung ist aber oft durch lange Fahrtwege, z. B. zum Universitätsklinikum, erschwert.

Der Digihub DECIDE („Decentralized digital Environment for Consultation, data Integration, Decision making and patient Empowerment“) entwickelt eine IT-Plattform, die versucht, eine Brücke zwischen Uniklinik, Arztpraxen, Krankenhäusern und Patient:innen zu schlagen, und Werkzeuge wie Telemedizin, künstliche Intelligenz und mobile Sensoren integriert. Hierbei werden Datenaustausch und die Zusammenarbeit bei Therapiefindung und -begleitung erleichtert sowie die gewonnenen Daten für die Forschung nutzbar gemacht.

Zur Therapiefindung wird eine telemedizinische Konsultation zwischen Ärzt:innen angeboten. Zudem steht für die Auswahl der Therapie ein Assistenzsystem zur Entscheidungsunterstützung bereit. Das mit künstlicher Intelligenz erweiterte Expertensystem beinhaltet Wissen aus Behandlungsleitlinien und aktuellen Studienergebnissen. Zudem ermöglicht es einen Vergleich mit ähnlichen Behandlungsfällen aus der Vergangenheit.

Zur Therapiebegleitung sind Handgelenksensoren (Wearables) und eine Smartphone-App für Patient:innen verfügbar. Mit den Messwerten der Sensoren, die an die App und von dort weiter an die IT-Plattform übermittelt werden, lässt sich der Therapiefortschritt überwachen. Um Patient:innen aktiv an der Behandlung zu beteiligen, stellt die entwickelte mobile App individualisierte Informationen bereit. Darüber hinaus können über Fragebögen Rückmeldungen der Betroffenen eingeholt werden.

Das DECIDE-System wird bei Patient:innen mit Lungenkrebs, Dickdarmkrebs oder Depressionen und ferner in einer zusätzlich angebotenen Bewegungstherapie angewendet. Die Bewegungstherapie wird mit der entwickelten Technik telemedizinisch aus der Ferne unterstützt. Die Teilnehmer:innen erhalten von Therapeut:innen einen Trainingsplan und Anleitungen auf ihr Smartphone, damit sie das Training zu Hause durchführen können. Über Wearables und Rückmeldungen via App wird die Aktivität überwacht. Das Assistenzsystem analysiert die Daten und schlägt, wenn nötig, den Therapeut:innen einen angepassten Trainingsplan vor.

Die bei Therapiefindung und -begleitung angefallenen Daten – gerade aus der regionalen Versorgung – werden nach Einwilligung der Patient:innen an das Datenintegrationszentrum der Universitätsklinik übertragen. Dort können sie Forschenden helfen, Krankheiten in der Breite zu verstehen und individuelle Ansatzpunkte für Therapien aufzuspüren.

### DISTANCE: Gezielte Vorsorge und Therapie nach intensivmedizinischer Behandlung

Nach einem intensivmedizinischen Aufenthalt zeigen viele Patient:innen unterschiedliche Symptome wie Atembeschwerden, körperliche Erschöpfung, Depressionsschübe oder Gedächtnisstörungen. Dieses Phänomen wird Post Intensive Care Syndrome (PICS) genannt und kann dazu führen, dass die Betroffenen erneut in die stationäre Behandlung müssen. Im Fokus von DISTANCE (Digital Smart Hub for Advanced Connected Care) steht die Verbesserung der intensivmedizinischen Nachsorge. Der Hub baut dabei auf den Vorarbeiten der Medizininformatik-Initiative auf – mit dem Ziel, Gesundheitsdaten aus der regionalen Versorgung für die Forschung verfügbar zu machen.

DISTANCE möchte durch die Entwicklung eines außeruniversitären digitalen Hubs, welchen die Partner gemeinsam aufbauen, eine Vernetzung von niedergelassenen Ärzt:innen, Kliniken und Patient:innen sicherstellen, um Daten für die Forschung und Versorgung zur Verfügung zu stellen.

Um die Funktionalität und den Nutzen der Datenausleitung zu demonstrieren, wurde in DISTANCE die PICOS-App entwickelt. Sie dient der therapeutischen Unterstützung für Patient:innen, die von der Intensivstation entlassen wurden. Die PICOS-App soll Neuaufnahmen vorbeugen, indem sie die medizinische Selbstfürsorge der Patient:innen in ihrem Alltag unterstützt. In regelmäßigen Abständen können die ehemaligen Intensivpatient:innen ihre Vitalwerte wie Herzfrequenz, Blutdruck, Blutzucker, körperliche Aktivität und Schlafdauer in die App eingeben und erhalten somit einen Überblick über ihren aktuellen Gesundheitszustand.

Daneben werden mit der App wertvolle Langzeitdaten gesammelt, welche die Versorgung und die Gesundheitsforschung unterstützen. Mit diesen Daten können Wissenschaftler:innen das PICS-Syndrom erforschen sowie noch unbekannte Zusammenhänge zwischen den Symptomen und den Krankheiten ehemaliger Intensivpatient:innen ermitteln. Die neuen Erkenntnisse sollen dabei helfen, eine Verschlechterung des Gesundheitszustandes individuell vorherzusagen.

Bei der Entwicklung der PICOS-App standen die Nutzer:innen im Vordergrund. Eine Vorstudie hat sichergestellt, dass die App intuitiv nutzbar ist und den Bedürfnissen ehemaliger Intensivpatient:innen entspricht. Somit leistet DISTANCE einen Beitrag zur Akzeptanz von digitalen Lösungen in der Medizin und motiviert Patient:innen, ihre Daten der medizinischen Forschung zur Verfügung zu stellen. Das Projekt wird derzeit zusammen mit regionalen Krankenhäusern, Arztnetzen und Pflegeeinrichtungen ausgerollt [16].

### LeMeDaRT: Optimierung des Datenflusses bei Prähabilitation, Atemwegserkrankungen und nichtalkoholischer Fettleber

Die Versorgung im ländlichen Raum kann durch frühzeitige geeignete Interventionen wesentlichen Einfluss auf Krankheitsverläufe haben. Der dafür notwendige bilaterale digitale Datenfluss zwischen der Versorgung vor Ort und der Spitzenmedizin ist jedoch bisher kaum etabliert. Neben der rein technischen Herausforderung einer sicheren Realtime-Datenintegration für die Zusammenarbeit mit Partnern in der Peripherie erweist sich vor allem die Abstimmung organisatorischer Abläufe auf sinnvolle und datengestützte Prozesse aktuell als arbeitsintensiv, da organisationale Interoperabilität noch weitgehend fehlt. Hier entwickelt LeMeDaRT („Lean Medical Data: the Right data at the right Time“) technisch-organisatorische Lösungen anhand von 3 Anwendungsfällen:

1. Prähabilitation: Patient:innen sollten eine schwere und komplexe Operation (OP) in einem bestmöglichen Zustand angehen. Mit Prähabilitation können Betroffene bereits vor dem Eingriff gezielt körperlich und psychisch gestärkt werden. Dies und auch eine individualisierte Begleitung nach der OP verbessern Heilungschancen und vermindern Komplikationen. Die dafür erforderliche sektorenübergreifende, interprofessionelle und datengestützte Kooperation sowie Koordination existieren bislang nicht.
2. Atemwegserkrankungen: Anhand der ersten Symptome einer Atemwegserkrankung kann die hausärztliche Praxis potenziell bedrohlich verlaufende Infektionserkrankungen wie COVID-19 oder Influenza nur schwer von banalen viralen Infekten unterscheiden. LeMeDaRT entwickelt digitale Lösungen – etwa in Kombination mit Testverfahren –, um die Versorgung an diesem Punkt handlungsleitend zu verbessern. So kann die Entscheidung für oder gegen Praxisbesuch, Antibiotikatherapie oder Krankenhauseinweisung unterstützt werden.
3. Prävention und Frühintervention der nichtalkoholischen Fettlebererkrankung: Eine nichtalkoholische Fettlebererkrankung bleibt lange asymptomatisch und damit unerkannt. Sie betrifft im mittleren Erwachsenenalter etwa jede fünfte Person. Um Frühstadien der Leberveränderung, die sich durch veränderte Ernährung und Lebensstil günstig beeinflussen lassen, von gefährlichen Pathologien besser zu unterscheiden, erprobt LeMeDaRT neue Präventionszugänge, die Verfahren aus der Spitzenmedizin in die Abläufe vor Ort zu integrieren.

LeMeDaRT erprobt hier das neue Arbeitsformat der *Innovationslabore*, in welchen Fachpersonen, klinische Expert:innen und Entwickler:innen in einem wenige Wochen dauernden Zyklus intensiv zusammenarbeiten und alle Ebenen der Interoperabilität adressieren.

### MIDIA-Hub: Bessere Nachsorge von Krebserkrankungen, optimierte Therapie gegen multiple Sklerose

Bei der Nachsorge von Krebserkrankungen und der Behandlung von Menschen mit multipler Sklerose (MS) entstehen in jedem Einzelfall über viele Jahre hinweg große Mengen unterschiedlicher Daten, z. B. Laborwerte, Bildgebung und andere diagnostische Befunde, Informationen zu therapeutischen Maßnahmen, aber auch von Patient:innen dokumentierte Daten zur Lebensqualität. In den Behandlungsprozess sind neben den Universitätskliniken auch nichtakademische Krankenhäuser, Rehakliniken und vor allem ambulante Versorger involviert.

MIDIA-Hub möchte durch die Entwicklung eines neuen Ärzteportals, das die Universitätskliniken Erlangen und München gemeinsam mit der Firma Siemens als Technologiepartner aufbauen, niedergelassene Ärzt:innen und Kliniken miteinander vernetzen. Zusätzlich wird ein Patientenportal etabliert, um die aktive Patientenmitwirkung und -mitentscheidung zu fördern. Zum Beispiel soll es den Menschen ermöglichen, eigene Daten digital zur Verfügung zu stellen und der Nutzung ihrer Daten zu Forschungszwecken zuzustimmen, ihre Einwilligung aber auch jederzeit mit wenigen Mausklicks zu widerrufen [17].

Der Anwendungsfall Krebserkrankungen umfasst Menschen, die an Brust- oder Prostatakrebs erkrankt sind. Die Nachsorge der Betroffenen kann sich über 10 Jahre und länger erstrecken. Auf Basis des Datenaustauschs über das Ärzteportal soll sichergestellt werden, dass die verschiedenen behandelnden Ärzt:innen ihre Strategien koordinieren und für jeden die bestmöglichen Entscheidungen treffen. Die Daten sollen aber auch der Forschung helfen, mithilfe intelligenter Analysen drängende Fragen zu beantworten: Warum erleidet beispielsweise die eine Brustkrebspatientin einen Rückfall, die andere aber nicht?

MS ist die häufigste neurologische Autoimmunerkrankung junger Erwachsener – und nicht heilbar. Es gibt jedoch zahlreiche Medikamente, die den Fortschritt der Erkrankung verlangsamen können. Um die therapeutischen Möglichkeiten bestmöglich zu nutzen, müssen Ärzt:innen die Behandlungspfade überwachen und stetig an die individuellen Verläufe der Erkrankung anpassen. In der Vielzahl individueller Krankheitsverläufe von Menschen mit MS soll es Forschenden durch das Portal ermöglicht werden, neue Ansatzpunkte zu entdecken, wann welches der Medikamente für welche Personen die vielversprechendste Option bietet.

### MiHUBx: Ein digitales Ökosystem zur Stärkung von medizinischer Forschung, Diagnostik und Therapie in Sachsen

Eine sektorenübergreifende und serviceorientierte Infrastruktur zur Förderung einer effektiven Zusammenarbeit und Vernetzung innerhalb verschiedener Akteur:innen und Initiativen im regionalen Gesundheitswesen bietet das Potenzial, die medizinische Forschung, Diagnostik und Therapie in der Region in ganz unterschiedlichen Kontexten zu stärken. Der Proof of Concept einer solchen Infrastruktur wird in MiHUBx (Medical Informatics Hub in Saxony) durch die Implementierung der folgenden Anwendungsszenarien realisiert:

1. Ophthalmologie trifft auf Diabetologie: Rund ein Viertel der Menschen mit Diabetes Typ 1 entwickelt eine Retinopathie, bei Typ 2 ist es ca. ein Achtel der Betroffenen. Die optimale Diagnose und Therapieentscheidung benötigt sowohl diabetologische als auch ophthalmologische Expertise, die Forschung entwickelt zunehmend versorgungsrelevante Biomarker. Neben einer besseren Kommunikation zwischen den Augenkliniken und Diabetolog:innen sollen die interdisziplinären Daten für die Entwicklung eines KI-basierten Entscheidungsunterstützungssystems zur optimalen Diagnose und Therapieentscheidung für bestmögliche individuelle Behandlungsoptionen genutzt werden.
2. Regionales Gesundheitsmonitoring und Versorgungsplanung: Eine möglichst präzise Vorhersage von infektions- und hitzebedingten Gesundheitsstörungen soll entwickelt werden, um eine effektive Planung von Ressourcen in der Akutversorgung (Notaufnahmen, Krankenhäuser) zu ermöglichen und um schnell und angemessen auf eine hohe Ressourcenauslastung bei regionalen und pandemischen Ereignissen reagieren zu können.
3. Digitale Workflow-Integration in der personalisierten Krebsmedizin: Die Entwicklung eines Portals für den reibungslosen und zeitnahen Datenaustausch, die digitale Einbindung behandelnder Ärzt:innen und aktive Einbeziehung von Patient:innen soll den Transfer von Forschungsergebnissen in die Versorgung beschleunigen.

Das Grundkonzept von MiHUBx ist dabei die nahtlose Integration der Methoden und Werkzeuge aus der MII und dem MIRACUM-Konsortium in die regionale Versorgungspraxis in Sachsen [18], um die Lücke zwischen medizinischer Forschung und Versorgung zu verringern.

## Diskussion

Die Digihubs decken eine Vielzahl unterschiedlicher Krankheitsbilder, außeruniversitärer Akteur:innen der Gesundheitsversorgung, Regionen und eingesetzter Methoden und Werkzeuge zur institutionsübergreifenden gemeinsamen Nutzung von Gesundheitsdaten ab. Die Herausforderungen in der konkreten Umsetzung lassen sich über die einzelnen Anwendungsszenarien hinweg generalisieren (s. auch Infobox). Sie finden sich auf allen Ebenen der Vernetzung, das heißt auf regulativer, technischer und prozessualer Ebene, aber auch in den Anforderungen und deren Priorisierungen.

Auf regulativer Ebene sehen wir eine große Heterogenität in den Bedürfnissen der Praxispartner. Einige Praxispartner wollten gerne nachträglich in den Konsortialvertrag aufgenommen werden, andere werden über einen Kooperationsvertrag integriert. Insgesamt stellten sich die Datenschutzanforderungen auch in den Universitätskliniken als sehr heterogen dar – dies ist zum einen durch die Landesdatenschutz- und Landeskrankenhausgesetze bedingt, zum anderen aber auch durch die sehr heterogene technisch-organisatorische Einbindung der Datenintegrationszentren. Die Vernetzung mit externen Datenquellen oder -empfängern ist alles andere als selbstverständlich. So stellte sich bei einigen die Datenausleitung aus Krankenhaus- oder Praxisinformationssystemen als besonders kritisch heraus, bei anderen wurde der Rückfluss der Daten als problematisch gesehen.

Eine besondere Herausforderung im Vergleich zu den bisherigen Projekten der MII ist der notwendigerweise personenidentifizierbare Austausch der Gesundheitsdaten, da diese ja (wenn auch prototypisch) im Behandlungskontext eingesetzt werden. Die aktuellen Konzepte des Datenteilens innerhalb der MII sind für die Nachnutzung der Daten in der Forschung ausgelegt – so steht im Mustertext der Patienteninformation explizit: „Alle unmittelbar Ihre Person identifizierenden Daten (Name, Geburtsdatum, Anschrift etc.) werden durch eine Zeichenkombination ersetzt (Codierung). Dieses interne Kennzeichen sowie Ihre damit verbundenen Patientendaten [falls zutreffend: und Biomaterialien] können dann nicht mehr direkt Ihrer Person zugeordnet werden“ [19]. Damit werden für die datenschutzkonforme intersektorale Datenübermittlung im Rahmen der Projekte zusätzliche Einwilligungen der Patient:innen benötigt.

In allen Digihubs besteht bei den Partnern großes Interesse und eine Bereitschaft, an den kooperativen, intersektoralen Netzwerken mitzuwirken. Da aber Forschung bei den Partnern anders als in den Universitätskliniken keine primäre Aufgabe ist, sind bei der Abwägung zwischen Aufwand und Nutzen die Kriterien der Partner andere als die der Universitätsmedizin. Pandemiebedingt wurde die Aufbau- und Vernetzungsphase der Medizininformatik-Initiative um ein Jahr verlängert, da die Projektpartner an den Unikliniken multiple Aufgaben zur Bewältigung der COVID-19-Pandemie, z. B. im Netzwerk Universitätsmedizin, übernehmen mussten [20]. Beim Start der Digihubs in der zweiten Hälfte 2021 hatten viele der Praxispartner auch in den Folgemonaten noch mit Ausbrüchen bei Patient:innen, aber auch beim Personal zu kämpfen. Für Lösungen, die zwar perspektivisch sinnvoll, aber (noch) nicht direkt in der Versorgung verankert sind, war das Zeitbudget entsprechend extrem beschränkt. Der allgemeine Fachkräftemangel sowohl in den Gesundheits- als auch in den IT-Berufen lässt oft kaum Spielraum, um neben der Aufrechterhaltung des Alltagsgeschäfts noch Ressourcen zu mobilisieren. Für die Praxispartner steht der konkrete Mehrwert viel direkter in Beziehung zum Aufwand – die gerade zu Beginn eines Forschungsprojekts typischen Ergebnisse wie Konzeptpapiere und wissenschaftliche Veröffentlichungen bieten für die Partner nur geringen Mehrwert.

Insgesamt ist bei der Berücksichtigung vieler verschiedener Stakeholder, die ja explizit in der Ausschreibung gefordert waren, die Balance der verschiedenen Stakeholderanforderungen immer wieder abzugleichen. So ist zum Beispiel der medizinische Nutzen des Datenflusses aus einer Rehaklinik zurück ins Krankenhaus eher gering, für die Forschung hingegen sind die Daten hochrelevant. Der Rückfluss erfordert noch einmal deutlich mehr technologisch regulative Abstimmung, mit einem zumindest für die Praxispartner nicht direkt erkennbaren Mehrwert.

Wie in Forschungsprojekten üblich, lassen sich die zur Antragsphase entwickelten Konzepte nicht unmodifiziert in der Praxis umsetzen, sondern es sind immer wieder neue Lösungsansätze gefordert. Hier muss gut zwischen Pragmatismus und zeitnaher Umsetzung auf der einen Seite und einer möglichst einfachen Anschlussfähigkeit an die Lösungen der MII auf der anderen Seite abgewogen werden [21]. Dies ist insbesondere auch im Austausch mit den Technologiepartnern zu beobachten. Durch die strikter gewordenen Anforderungen im Medizinprodukterecht ist eine prototypische Umsetzung, z. B. die Implementierung von FHIR-Schnittstellen oder die Anbindung an andere Systeme, oft mit zeitaufwändigen Prozessen zur Erhaltung der Zertifizierung verbunden, zudem erfordern viele Anwendungsfälle als Medizinprodukt zertifizierte Software.

Die unterschiedlichen Standardisierungsaktivitäten der MII einerseits und der Kassenärztlichen Bundesvereinigung (KBV) und der Gematik GmbH andererseits zeigen trotz des Austauschs zwischen den Initiativen teilweise Unterschiede und Inkompatibilitäten. Dies ist in der Praxis aufgrund der aktuell nur sehr geringen Verfügbarkeit der Standards der Telematikinfrastruktur 2.0 kein akutes technisches Problem, jedoch ist es für die Technologiepartner unattraktiv, verschiedene Standards zu implementieren.

Der extrem heterogene Digitalisierungsgrad der an den Digihubs beteiligten Gesundheitseinrichtungen stellt insofern ein relevantes Problem dar, als Daten nur selten aus Primärsystemen übernommen werden können. Dies bedeutet an vielen Stellen eben doch die im Rahmen der Digitalisierung des Gesundheitssystems vieldiskutierte „Doppeldokumentation“ – hier in der lokalen Akte und in der digitalen Infrastruktur der Digihubs. Unter diesen Bedingungen wird die Notwendigkeit von nutzer:innenzentriertem Design und Technologieakzeptanz evident. Partizipative Entwicklung sowie effizient und intuitiv zu bedienende Oberflächen scheinen bisher bei medizinischen Dokumentationssystemen kaum eine Rolle zu spielen – dabei macht es für die Akzeptanz einer Maßnahme einen großen Unterschied, ob man z. B. für die Signatur auf einem Rezept eine Sekunde oder eine Minute benötigt.

Erfolgreiche Lösungen der Digihubs können zwar grundsätzlich die Anforderungen an Systeme der medizinischen und pflegerischen Dokumentation erfüllen, aber unter den aktuellen Förderbedingungen kann ein nachhaltiger und langfristiger Betrieb der Infrastruktur nicht gewährleistet werden – was den Mehrwert für die Praxispartner dann wieder deutlich reduziert. Auch bei der Analyse von Erfolgsfaktoren und Barrieren bezüglich der Nutzung von Patientenportalen stellten sich die genannten Aspekte als relevant heraus [17].

Der dauerhafte Transfer von erfolgreichen Lösungen in die Praxis wird auch dadurch besonders erschwert, dass der erste Gesundheitsmarkt (die „klassische“ Gesundheitsversorgung, finanziert v. a. durch Kranken- und Pflegeversicherung) sehr stark reguliert ist. Wenn die Nutzung von digitalen Lösungen nicht in der Regelversorgung verankert ist, sind diese kaum profitabel zu betreiben – selbst wenn sie nachweislich einen Nutzen für die Gesundheitsversorgung haben. Für diese digitalen Lösungen tragfähige Nachhaltigkeitskonzepte zu entwickeln, stellt eine völlig andere Herausforderung dar als für Lösungen, die ausschließlich in der Forschung eingesetzt werden. Die starren Mechanismen des ersten Gesundheitsmarktes machen es extrem schwer, innovative Lösungen auch über die Projektförderung hinaus marktreif zu entwickeln und dann tatsächlich erfolgreich zu etablieren – hier bestimmen eben nicht Angebot und Nachfrage, sondern Kosten und Erstattungsfähigkeit den Markt. Die Telematikinfrastruktur 2.0 mit einem grundsätzlich modularen und interoperablen Systemansatz ist zwar auf einem guten Weg, da sie technologisch die Möglichkeit für die Integration von Spezialanwendungen eröffnet, sie ändert aber nichts an den Finanzierungsmechanismen.

Die Vernetzung der Digihubs in den Strukturen der MII, inklusive der Vertretung im Nationalen Steuerungsgremium, fördern eine kontinuierliche Abstimmung und die gemeinsame Entwicklung von Lösungsansätzen. Ein gemeinsames Positionspapier empfiehlt folgende Maßnahmen zu priorisieren: Vereinheitlichung der Datenschutzregelungen, Maßnahmen zur Steigerung von Akzeptanz und Vertrauen in Digitalisierung und gemeinsame Datennutzung sowie die Schließung des Transfer-Gaps durch geeignete Förderinstrumente [22].

## Fazit

Die Digihubs bauen auf den Pionierarbeiten der MII auf, sind aber durch vielschichtige Erweiterungen der Anwendungsszenarien in die regionale Versorgung ihrerseits Pioniere in der digitalen Transformation des Gesundheitssystems. Gesetzesinitiativen wie das Gesundheitsdatennutzungsgesetz als Implementierung des Europäischen Gesundheitsdatenraums können zukünftig Hürden im Bereich des Datenschutzes und der Interoperabilität senken und neue Möglichkeiten des Datenteilens eröffnen. Viele der in der Umsetzung der Digihubs erfahrenen Schwierigkeiten haben komplexe Ursachen und erfordern weitergehende Maßnahmen der Förderung der digitalen Transformation, um den Transfer-Gap zu verringern und die in den Digihubs oder ähnlichen Initiativen erfolgreich entwickelten Konzepte, Methoden und Lösungen dauerhaft für eine Verbesserung der Gesundheitsversorgung zu etablieren.

### Infobox Gemeinsame Herausforderungen der Digihubs

*Nichtuniversitäre Partner*:

- Andere Incentives als akademische Partner
- Andere Priorisierung von Datenaustausch
- Forschung keine primäre Aufgabe
- Fachkräftemangel und Gesundheitskrisen
- Mangelnde Digitalisierung
- Mangelnde Usability

*Rechtliche Rahmenbedingungen*:

- Heterogenes Datenschutzrecht
- Heterogene Datenschutzbedenken
- Austausch identifizierender Daten notwendig
- Medizinproduktanforderungen bei Prototypen

*Transfer in die Praxis*:

- Mechanismen primärer Gesundheitsmarkt
- Projektförderung nur bis Prototyp
- Dynamik Digitalisierung im Gesundheitswesen

## References

[1] Semler SC, Wissing F, Heyder R (2018). German medical Informatics initiative. Methods Inf Med.

[2] Wilkinson MD, Dumontier M, Aalbersberg Ij J (2016). The FAIR Guiding Principles for scientific data management and stewardship. Sci Data.

[3] Bild R, Bialke M, Buckow K (2020). Towards a comprehensive and interoperable representation of consent-based data usage permissions in the German medical informatics initiative. BMC Med Inform Decis Mak.

[4] Ganslandt T, Boeker M, Löbe M et al (2018) Der Kerndatensatz der Medizininformatik-Initiative—Interoperable Spezifikation am Beispiel der Laborbefunde mittels LOINC und FHIR. mdi—Forum der Medizin, Dokumentation und Medizin-Informatik 20:113–117. https://www.medizininformatik-initiative.de/sites/default/files/2018-07/2018-03_mdi_Der%20Kerndatensatz%20der%20Medizininformatik-Initiative%20Ein%20Schritt%20zur%20Sekund%C3%A4rnutzung%20von%20Versorgungsdaten%20auf%20nationaler%20Ebene.pdf. Zugegriffen: 26. Febr. 2024

[5] Prokosch H-U, Gebhardt M, Gruendner J (2023). Towards a national portal for medical research data (FDPG): vision, status, and lessons learned. Stud Health Technol Inform.

[6] Internetredaktion RBL Richtlinie zur Förderung von Zuwendungen für „Digitale FortschrittsHubs Gesundheit“ im Förderkonzept Medizininformatik – DLR Gesundheitsforschung [Internet]. Deutsche Zentrum für Luft und Raumfahrt e. V. – DLR Gesundheitsforschung. https://www.gesundheitsforschung-bmbf.de/de/10580.php. Zugegriffen: 26. Febr. 2024

[7] Krefting D (2021) Digitale Transformation: Neues Tempo nutzen. Deutsches Ärzteblatt 118:A-2437 / B‑2000. https://www.aerzteblatt.de/archiv/222696/Digitale-Transformation-Neues-Tempo-nutzen. Zugegriffen: 26. Febr. 2024

[8] Lypp L (2021) Deutscher Bundestag – Nachholbedarf bei der Digitalisierung im Gesundheitswesen. https://www.bundestag.de/dokumente/textarchiv/2021/kw28-pa-pandemie-digitalisierung-851226. Zugegriffen: 26. Febr. 2024

[9] German Science And Humanities Council (2022). Digitalisierung und Datennutzung für Gesundheitsforschung und Versorgung – Positionen und Empfehlungen | Positionspapier.

[10] Deutscher Bundestag (2023) Anhörung zum Gesundheitsdatennutzungsgesetz (GDNG). https://www.bundestag.de/ausschuesse/a14_gesundheit/oeffentliche_anhoerungen/974686-974686. Zugegriffen: 26. Febr. 2024

[11] Herzstiftung D (2023) Deutscher Herzbericht 2022. https://www.dgpk.org/wp-content/uploads/DHB22-Herzbericht-2022.pdf. Zugegriffen: 26. Febr. 2024

[12] Sommer KK, Amr A, Bavendiek U (2022). Structured, harmonized, and interoperable integration of clinical routine data to compute heart failure risk scores. Life.

[13] Haarbrandt B, Schreiweis B, Rey S (2018). HiGHmed—an open platform approach to enhance care and research across institutional boundaries. Methods Inf Med.

[14] Hofmann A-L, Schmidt J, Selig U (2023). Development of a digital hub for improving emergency stroke care and introducing quality indicators to evaluate its impact: the CAEHR project. ESOC 2023 abstract book.

[15] Schmidt J, Hofmann A‑LJ-PR et al (2023) Requirements when implementing an interface for improved emergency stroke care: Process Evaluation of the emergency use case within the CAEHR project. In: DGEpi 2023 Abstract Book. Würzburg: Deutsche Gesellschaft für Epidemiologie. https://2023.dgepi.de/wp-content/uploads/2023/09/Abstractbook__DGEpi2023_.pdf. Zugegriffen: 26. Febr. 2024

[16] Molinnus D, Kurth A, Lowitsch V (2023). Towards an advanced digital infrastructure within the non-university sector demonstrated by the PICOS app. Stud Health Technol Inform.

[17] Leb I, Magnin S, Prokosch H-U, Boeker M (2021). Patient portals: objectives, acceptance, and effects on health outcome—A scoping review of reviews. Stud Health Technol Inform.

[18] Prokosch H-U, Acker T, Bernarding J (2018). MIRACUM: medical Informatics in research and care in university medicine: a large data sharing network to enhance translational research and medical care. Methods Inf Med.

[19] Medizininformatik-Initiative (2020) Mustertext zur Patienteneinwilligung Version 1.6 d. https://www.medizininformatik-initiative.de/sites/default/files/2020-04/MII_AG-Consent_Einheitlicher-Mustertext_v1.6d.pdf. Zugegriffen: 26. Febr. 2024

[20] Heyder R (2023). Das Netzwerk Universitätsmedizin: Technisch-organisatorische Ansätze für Forschungsdatenplattformen. Bundesgesundheitsblatt Gesundheitsforschung Gesundheitsschutz.

[21] Koch M, Richter J, Hauswaldt J, Krefting D (2023). How to make outpatient healthcare data in Germany available for research in the dynamic course of digital transformation. Stud Health Technol Inform.

[22] Medizininformatik-Initiative (2023) Positionspapier zum politischen Abend der Digitalen FortschrittsHubs. https://www.medizininformatik-initiative.de/sites/default/files/2023-09/2023-09-06_Positionspapier%20DigiHubs_final.pdf. Zugegriffen: 26. Febr. 2024

